# Infant Food Hygiene and Childcare Practices in Context: Findings from an Urban Informal Settlement in Kenya

**DOI:** 10.4269/ajtmh.19-0279

**Published:** 2019-11-18

**Authors:** Jane Awiti Odhiambo Mumma, Oliver Cumming, Sheillah Simiyu, Alexandra Czerniewska, Rose Evalyne Aseyo, Damaris Nelima Muganda, Emily Davis, Kelly K. Baker, Robert Dreibelbis

**Affiliations:** 1 Center of Research, Great Lakes University of Kisumu, Kisumu, Kenya;; 2 Department of Disease Control, London School of Hygiene and Tropical Medicine, London, United Kingdom;; 3 Department of International Health—Social and Behavioral Interventions, Johns Hopkins Bloomberg School of Public Health, Baltimore, Maryland;; 4 Department of Occupational and Environmental Health, College of Public Health, University of Iowa, Iowa City, Iowa

## Abstract

Complementary food hygiene is important to reduce infant exposures to enteric pathogens; however, interventions to improve food hygiene in low- and middle-income countries often ignore the larger context in which childcare occurs. In this study, we explore on observational and qualitative information regarding childcare in an informal community in Kenya. Our findings demonstrate that behaviors associated with food contamination, such as hand feeding and storing food for extended periods, are determined largely by the larger social and economic realities of primary caretakers. Data also show how caregiving within an informal settlement is highly dynamic and involves multiple individuals and locations throughout the day. Findings from this study will help inform the development and implementation of food hygiene interventions in informal urban communities.

In many low- and middle-income countries (LMIC), complementary foods given to infants during the critical weaning period can be highly contaminated,^1–3^ and contaminated weaning foods are a neglected source of enteric pathogen exposure.^4^ Complementary food hygiene interventions have shown significant reductions in infant food contamination and improved behaviors.^1,2,5^ However, there is limited evidence to support the design and delivery of effective, scalable food hygiene interventions,^6^ particularly in urban neighborhoods. Recent studies have also given limited attention to the social context in which infant food preparation, food hygiene, and childcare occur. This study aimed to understand childcare practices in a low-income community in East Africa and understand the contextual factors that influence caregiver food hygiene practices related to complementary food hygiene.

The study was conducted in an informal settlement in Kisumu, Kenya. Over half of Kisumu’s inhabitants live in dense, informal neighborhoods with limited public health infrastructure.^7,8^ With assistance from 15 local community health volunteers (CHVs), we identified four to five households with children aged 3–9 months in each CHV’s catchment area. Our purposive sampling strategy ensured that participants reflected the range of environmental conditions in the neighborhood. A total of 70 households were identified, of which 57 agreed to participate in the study. Households were recruited in two rounds, approximately 1 month apart. In each household, the index child and the primary caregiver, defined as the person who was directly responsible for the index child, were identified. Structured observations and in-depth interviews captured data on childcare, food preparation, and feeding practices. Observations lasted between 1.5 and 6 hours (average 3 hours) and were completed either in the morning or in the afternoon. Data were collected by students (four women and two men) enrolled in a bachelor’s degree program in Public Health, who were trained on the study protocol. Quantitative analysis of caregiver handwashing during the first round of observations is described in a previous publication.^9^ In the second round of observations, caregivers were asked to demonstrate specific infant food preparation and handling practices. In-depth interviews lasted for approximately 30 minutes and were completed immediately after the observation. Interviews explored environmental, cognitive, and cultural determinants of childcare and food hygiene behaviors.

Participating caregivers included 29 mothers and 28 non-maternal caregivers (Table 1). Ten percent of respondents reported that they were the sole caregivers of the index children. Half of all the index children had three or more routine caregivers (Figure 1). Childcare occurred in a variety of locations. Only 32% of caregivers reported that the child spent its full day at home; approximately 30% of respondents said that they took the child to work during the day, including local retail shops and food stalls by the highways.

**Table 1:** Primary caregivers who participated in the study

Caregiver	% (*n*)
Aunt	14 (8)
Grandmother	14 (8)
Domestic help	12 (7)
Mother	51 (29)
Mother’s friend	02 (1)
Neighbor	12 (7)

**Figure 1.**
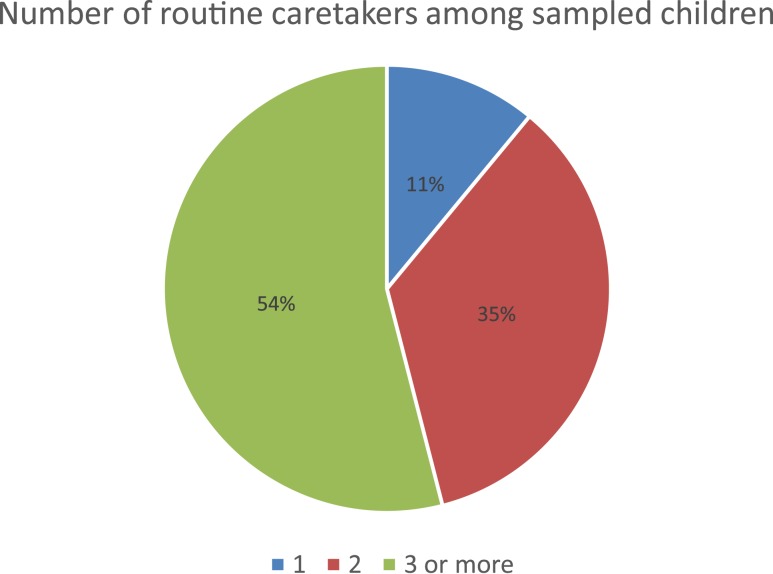
Number of routine caretakers among sampled children. This figure appears in color at www.ajtmh.org.

Two of the 57 children were exclusively breastfed. Complementary feeding started as early as at the age of 3 months. Reasons for starting earl included mothers not producing enough breast milk and they needed to work during the day. In addition to breast milk, the most common child foods were cow milk, porridge, mashed potatoes, *ugali* (cornmeal porridge), and cooked bananas.

In 37 of 57 households, caregivers reported that food was prepared on a charcoal or paraffin stove within a one-room home; only 16 households (28%) had a separate kitchen within the house. Others cooked outside the house on firewood stoves. Infant food was usually prepared when family meals were prepared, typically once or twice per day.

Several caregivers reported preparing infant food in the morning and leaving it with other caregivers while at work, requiring food storage. Milk and porridge were stored in a thermos or flask. Solid and semisolid foods (mashed potatoes, ugali, and bananas) were typically stored in a *sufuria* (saucepan), on a plate, or in a container. Sixty-three percent of caregivers said food prepared in the morning lasted all day. Only half of the respondents reported reheating food before serving to the child. Reheating was mostly focused on making food more palatable to children—caregivers reported reheating food “to make the food warm for the baby” or “to avoid feeding the baby cold food.” No caregivers were observed reheating food to boil. Roughly, a quarter of all the caregivers who were feeding children solid or semisolid foods (12 of 55, 22%) reported adding other ingredients such as water or milk to food after reheating.

Caregivers described multiple styles of child feeding. A feeding bottle, a cup, and/or the hand were all used to feed children, and multiple feeding styles were used within the same meal. Hand feeding of porridge and semisolid food involved *gargling*; the caregiver would place porridge in their palm, open the infant’s mouth, and push the food into the infant’s mouth bit by bit until it is finished. Caregivers reported that gargling enables the infant to eat more porridge in a short period of time. For solid foods, caregivers placed the food on a plate and used a spoon to feed the infant or fed the infant with their fingers. All caregivers reported handwashing before food preparation and feeding and that they used only clean utensils. There were no differences in reported food hygiene or feeding behaviors between maternal and non-maternal caregivers.

Our findings have implications for the design and delivery of food hygiene interventions targeting urban populations in LMIC and demonstrate how efforts to improve food hygiene behaviors need to be understood within context. Critical behaviors are necessary to maintain adequate food hygiene in domestic settings, including washing hands with soap, cooking food thoroughly, and storing food at safe temperatures.^10^ Complementary food hygiene practices found in our study are similar to those described in other resource- and infrastructure-constrained communities in LMIC.^1,2,5,6,11^ Although caregivers reported frequent handwashing and using clean utensils before preparing the child’s food and child feeding, this was not supported by observational data from the same population.^9^ The large proportion of caregivers that rely on hand feeding infants further increases the risk of pathogen exposure, given low rates of hand hygiene; however, hand feeding was practiced to minimize both the time required to feed the child and the potential for the child to waste food.

Storing complementary foods for extended periods of time at ambient temperatures is associated with increased bacterial contamination, especially in tropical climates such as those in East Africa.^3^ Infant food was prepared in parallel with food preparation for the larger family and timing determined by caretaker’s economic responsibilities outside of the home. Because of employment demands and reliance on charcoal and paraffin stoves, reheating food—a common behavioral target for food hygiene interventions^1,2,5^—requires time and economic resources that families do not have. Many interventions focus on changing food hygiene behaviors in the immediate home environment.^5^ Our findings show that in an informal urban environment, childcare and child feeding can occur at multiple locations in and around the home and the neighborhood.

Multiple caregiver arrangements with frequent changes in care over time are commonly observed in low-income communities.^12,13^ However, many community-based food hygiene interventions explicitly target a single—usually maternal—caregiver.^5,6^ The association between multiple caregivers and exposure to enteric pathogens in young children living in urban poor communities requires investigation and intervention strategies that target the range of formal and informal caregiving practices.

Complementary feeding started before the current national and international guidelines for exclusive breastfeeding (EBF)^14,15^ but at similar ages to those reported in other Kenyan informal settlements.^16^ In our study, early introduction of complimentary foods was determined by perceived quantity of maternal milk production and maternal employment. Children of mothers who are undernourished often need supplementary foods to maintain optimal growth,^17^ and studies have documented lower volumes of breast milk production in communities with poor living conditions.^18^ Data on the impact of maternal employment and improved infant feeding, however, are mixed. A recent multicountry analysis found no association between EBF and maternal employment at 6 months.^19^ Our findings suggest that maternal employment—which often drives the need to store food for long periods and feeding and caring for children away from the home—may be a determinant of food contamination and warrants further investigation.

Lack of random sampling is a potential limitation of our study, and sampling procedures may have resulted in a nonrepresentative sample of households. Aware of our affiliation with a research and public health institution, caregivers may have provided biased responses. However, our findings provide valuable insights into caregiving and food hygiene in low-income urban neighborhoods. Caretaking in this informal, urban environment is highly dynamic, suggesting that interventions should target not just immediate members of the household. Furthermore, the caretakers’ ability to successfully follow recommended food hygiene practices may be limited by the socioeconomic context in which childcare occurs. Interventions that are sensitive to the temporal, financial, and spatial constraints faced by inhabitants of urban informal settlements are needed. To achieve acceptable complementary food hygiene standards in these neighborhoods may be contingent on improvements in large-scale infrastructure and in the material living conditions of respondents.
